# A single institution, cross-sectional study on medical student preferences for collaborators in interprofessional education

**DOI:** 10.1186/s12909-023-05006-5

**Published:** 2024-02-23

**Authors:** Emily C. Goins, Margaret Coates, Alexander Gordee, Maragatha Kuchibahtla, Kathleen Waite, Erin Leiman

**Affiliations:** 1 Department of Emergency Medicine, Northwestern University, 250 East Huron, Chicago, IL 60611 USA; 2 Department of Dermatology, University of North Carolina, Chapel Hill, NC USA; 3 Department of Biostatistics and Bioinformatics, Duke University, Durham, NC USA; 4 Department of Medicine, Duke University, Durham, NC USA; 5 Department of Emergency Medicine, Duke University, Durham, NC USA

**Keywords:** Medical education, Interprofessional education, Student preference, Medical student, Emergency department

## Abstract

**Background:**

While the importance of interprofessional education in medical training has been well-established, no specific framework has been used uniformly or shown to be most effective in the creation of interprofessional education (IPE) sessions. Further, prior studies have demonstrated that students have preferences for the design of these experiences. In this study, we sought to understand medical student preference for interprofessional teammates and motivations for this choice.

**Methods:**

In this single-institution, cross-sectional analysis of the Duke IPE Clinic, participating students from September 2019–March 2020 completed a voluntary electronic survey that queried preferences for which health professions students (Doctor of Physical Therapy (DPT), Accelerated Bachelor of Science in Nursing (ABSN), Nurse Practitioner (NP), Pharmacy, and Physician’s Associate (PA)) they would want to work with, and the motivating reason. Preferences and reasons were compared between first-year medical students (MS1s) and third- and fourth-year medical students (MS3s/MS4s).

**Results:**

In total, 132 students participated. We found that MS1s most preferred interprofessional teammates with a more similar area of study (PA, NP), whereas MS3s/MS4s most preferred classmates with a less similar area of study (pharmacy, DPT, ABSN). MS1 students frequently selected their first-choice preference because the profession seemed most similar, while MS3/MS4 students often selected their first-choice preference because the profession seemed most different.

**Conclusions:**

Medical students earlier in training have more interest in working with professions they view as similar whereas senior students prefer to work with professions they view as more different. This information is important for designing educational IPE opportunities.

## Background

As the United States population ages and life expectancy continues to prolong with rapid medical advancement, the health system is faced with ever-more complex patients. As a result, there is an increasing need for collaborative practice among healthcare professionals. Effective healthcare interprofessional collaboration contributes to increased patient satisfaction, reduces medical errors, improves cost-effectiveness, and enhances patient care and safety [1, 2]. It has also been shown to improve healthcare providers’ job satisfaction due to reduced workloads, assistance with approaching difficult patient issues, and increased awareness of available resources [3]. However, studies have shown that multiple barriers to effective interprofessional communication exist, especially in high-acuity settings like the Emergency Department (ED), partially due to lack of formal training [4].

Therefore, there has been a movement in medical education to provide students with the core competencies required to work effectively in multidisciplinary teams. According to the World Health Organization, interprofessional education (IPE) is where students from one or more professions learn about, from, and with each other [5], with the goal of practicing pertinent skills with other trainees prior to entering the workplace. When institutions design IPE experiences, the learning goals of these activities should be drawn from pre-existing frameworks, which have been outlined several times by different nations, including in the International Consensus Statement on the Assessment of Interprofessional Learning Outcomes [6, 7]. These core competencies include: (1) Values and Ethics, (2) Roles and Responsibilities for Collaborative Practice, (3) Interprofessional Communication, and (4) Teamwork and Team-Based Care [8].

In the literature, interprofessional learning opportunities have included a wide variety of environments including clinical simulations, panel presentations, interactions with patients in structured clinical settings, anatomy dissections, and formal didactic teaching [2]. No specific learning theory or explicit teaching framework has been used uniformly or shown to be most effective in the creation of IPE sessions [2]. However, across many models of IPE, studies have shown that health care professional students, including medical students, report that they enjoy these sessions, gain respect and insight into different health care professional experiences and perspectives, and learn the importance of as well as the skills required for collaborative models of care [1–3, 9–11].

Previous studies have described student perception of several aspects of IPE experiences, such as preference for busier clinical sites, experiences with more direct patient care, and educational sessions where there is prior identification of roles and goals for each student [12–14]. In this novel, single-institution retrospective analysis, we sought to understand interprofessional education preferences of medical students from the Duke University Doctor of Medicine Program, and determine whether there was a difference in stated preferences between the first-year medical students (MS1s), and the third- and fourth-year medical students (MS3s/MS4s).

## Methods

### Duke interprofessional education clinic

The Duke Interprofessional Education (IPE) Clinic is a clinical learning experience that is staffed by varied health professions faculty members and students, and provides care to low-acuity patients presenting to the ED. The professions involved include Doctor of Physical Therapy (DPT), Accelerated Bachelors of Science in Nursing (ABSN), Nurse Practitioner (NP), Pharmacy, and Physician’s Associate (PA). DPT professionals treat disease and injury with hands-on care and prescribed exercises. ABSNs, who are nurses, provide bedside care to patients, giving medications and carrying out orders prescribed by doctors. PA and NP providers are classified as advanced practice providers, who can carry out many of the same tasks as doctors including seeing patients and prescribing medications, however they must do so under the supervision and license of a medical doctor. In our IPE Clinic, the composition of the student teams varies with each clinical session and does not include all professions in any one session.

In the Duke University Doctor of Medicine program, all MS1s participate in at least one four-hour IPE clinic session. MS2s do not participate in the IPE Clinic because they are completing their mandatory clinical rotations and there is no time in the curriculum to attend clinic, thus they are not included in the structure of the clinic nor in the data collected in this study. Senior MS3/MS4s return to the IPE Clinic after they have already completed their core clerkships, which at Duke is in their second year of study. MS3/MS4s can participate in several clinic sessions. Participation in the IPE clinic is optional for MS3/MS4s.

### Survey data collection

**Fig. 1:**
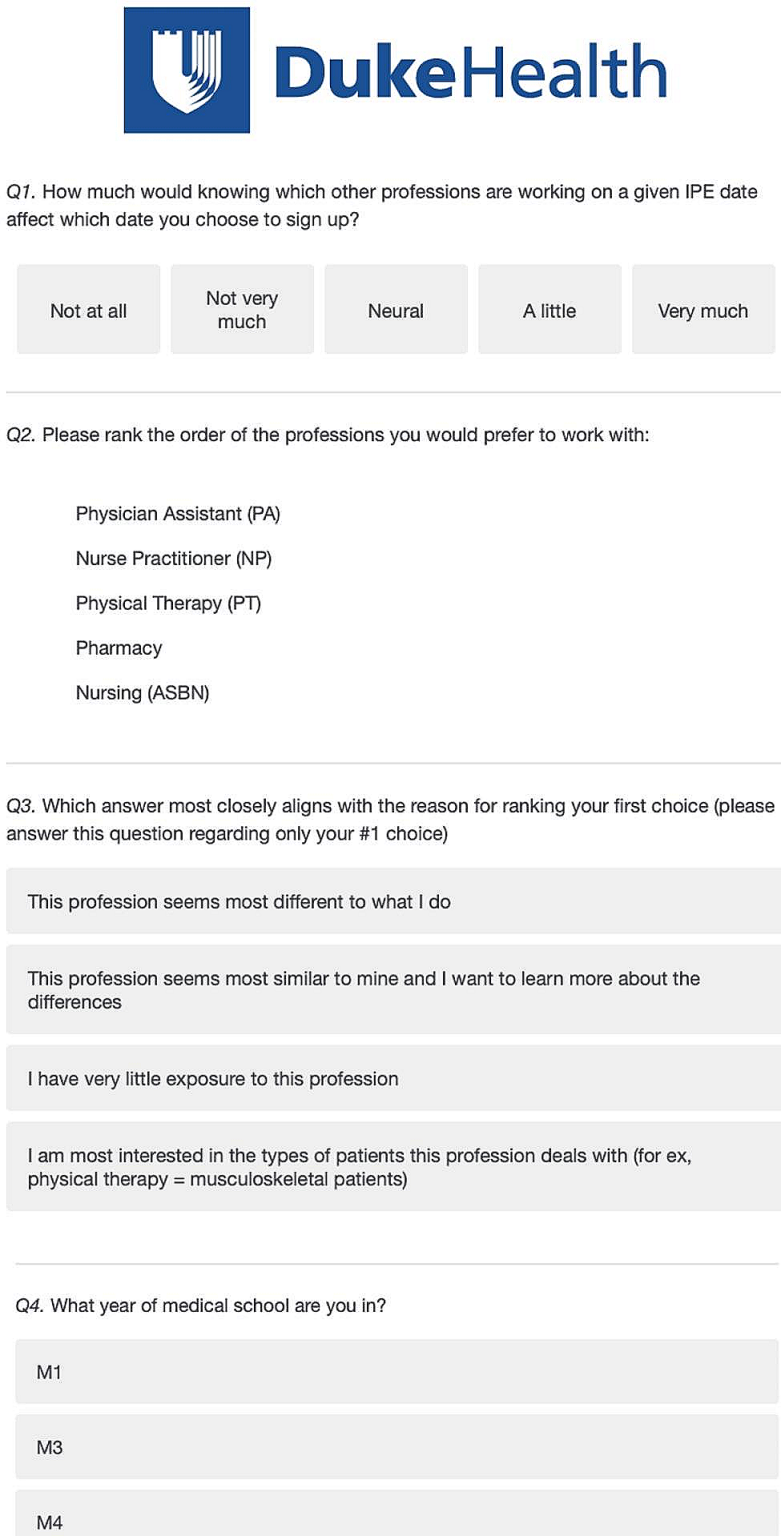
Voluntary electronic survey for data collection

Between February and March 2020, Doctor of Medicine (MD) students who had participated in the Duke IPE clinic between September 2019-March 2020 were surveyed on their preferences of which health professions students (DPT, ABSN, NP, Pharmacy, and PA) they would like to work with, and why. Voluntary electronic student surveys were sent out to all participating medical students. The questions are shown in Fig. 1.

### Statistical Analysis

Participant rankings and first choice preferences, along with reason for first choice ranking were summarized using frequency with percentage or mean with standard deviation and median with interquartile range where appropriate (Table 1). Results were presented and interpreted by year of program (MS1s vs. MS3/MS4s).

The proportions of students choosing each profession as their first choice and the reason for choosing the first ranked option were each compared between MS1 and MS3/MS4 students using Fishers exact tests. A secondary comparison of the proportion of students choosing a profession more similar to MD versus less similar to MD between MS1s and MS3/MS4s was performed using a Fishers exact test. In this study, the professions were grouped into clinical providers who can diagnose and prescribe medications and treatments, often autonomously, i.e., MD, PA, and NP, and those professions that provide a separate and distinct type of medical care that is often performed alongside the clinical provider, i.e. PT, nursing, and pharmacy. Thus, PA and NP students were considered more similar to MD students, while DPT, ABSN, and pharmacy students were considered less similar. All analyses were performed using R version 4.2.2.

## Results

### Student Preferences for Fellow IPE Participants

A total of 132 students responded to the voluntary surveys, which was 34.0% of all 388 students who received the surveys. Of these 132 students, 46 (34.8%) were MS1s, and the remaining 86 were MS3s/MS4s. Of the 132 respondents, 124 (93.9%) students completed the preference portion of the survey (43 (34.7%) MS1 and 81 MS3/4).

For first choice preferences, 16/43 (37.2%) MS1 students would most prefer to work with PA students in the IPE clinic, while only 17/81 (21.0%) MS3/MS4 students most prefer to work with PA students (Table 1). Conversely, 5 (11.6%) MS1s most prefer to work with pharmacy students, while 22 (27.2%) MS3/MS4s most prefer to work with pharmacy students. Similarly, MS1s more often selected NP students as their first choice than MS3/MS4s, whereas MS3/MS4s more often had DPT students as their first choice than MS1s. A roughly equal number of MS1s and MS3s/MS4s most preferred to work with ABSN students. No statistically significant difference was found in the overall first-choice preference of MS1 and MS3/4 students (p = 0.087).

Preferences were grouped based on the similarity of the degree type to an MD, with PA and NP students being considered more similar to MD students and pharmacy, DPT, and ABSN students being considered less similar to MD students. Comparing the first choice based on these groupings, it was found that 23 (53.5%) MS1s most preferred classmates with a more similar area of study, whereas 57 (70.4%) MS3/MS4s most preferred classmates with a less similar area of study. The proportion of students preferring classmates with a more similar area of study was significantly different between MS1 and MS3/4 students (p = 0.012).

**Table 1:** Summary of survey response, by year of study

	MS1 (N = 46)	MS3/MS4 (N = 86)	Overall (N = 132)
**First Choice**			
ABSN	9 (20.9%)	18 (22.2%)	27 (21.8%)
NP	7 (16.3%)	7 (8.6%)	14 (11.3%)
PA	16 (37.2%)	17 (21.0%)	33 (26.6%)
Pharmacy	5 (11.6%)	22 (27.2%)	27 (21.8%)
DPT	6 (14.0%)	17 (21.0%)	23 (18.5%)
Missing	3	5	8
**First Choice Similar to MD? (PA or NP)**			
No	20 (46.5%)	57 (70.4%)	77 (62.1%)
Yes	23 (53.5%)	24 (29.6%)	47 (37.9%)
Missing	3	5	8
**Second Choice**			
ABSN	11 (25.6%)	11 (13.6%)	22 (17.7%)
NP	14 (32.6%)	23 (28.4%)	37 (29.8%)
PA	9 (20.9%)	10 (12.3%)	19 (15.3%)
Pharmacy	5 (11.6%)	17 (21.0%)	22 (17.7%)
DPT	4 (9.3%)	20 (24.7%)	24 (19.4%)
Missing	3	5	8
**Third Choice**			
ABSN	12 (27.9%)	16 (19.8%)	28 (22.6%)
NP	8 (18.6%)	15 (18.5%)	23 (18.5%)
PA	9 (20.9%)	15 (18.5%)	24 (19.4%)
Pharmacy	9 (20.9%)	20 (24.7%)	29 (23.4%)
DPT	5 (11.6%)	15 (18.5%)	20 (16.1%)
Missing	3	5	8
**Fourth Choice**			
ABSN	7 (16.3%)	17 (21.0%)	24 (19.4%)
NP	9 (20.9%)	18 (22.2%)	27 (21.8%)
PA	5 (11.6%)	25 (30.9%)	30 (24.2%)
Pharmacy	8 (18.6%)	9 (11.1%)	17 (13.7%)
DPT	14 (32.6%)	12 (14.8%)	26 (21.0%)
Missing	3	5	8
**Fifth Choice**			
ABSN	4 (9.3%)	19 (23.5%)	23 (18.5%)
NP	5 (11.6%)	18 (22.2%)	23 (18.5%)
PA	4 (9.3%)	14 (17.3%)	18 (14.5%)
Pharmacy	16 (37.2%)	13 (16.0%)	29 (23.4%)
DPT	14 (32.6%)	17 (21.0%)	31 (25.0%)
Missing	3	5	8
**Reason**			
I am most interested in the types of patients this profession deals with (for ex, physical therapy = musculoskeletal patients)	2 (4.5%)	1 (1.2%)	3 (2.4%)
I have very little exposure to this profession	13 (29.5%)	23 (27.7%)	36 (28.3%)
This profession seems most different to what I do	7 (15.9%)	35 (42.2%)	42 (33.1%)
This profession seems most similar to mine and I want to learn more about the differences	22 (50.0%)	24 (28.9%)	46 (36.2%)
Missing	2	3	5

M1 = first year medical student, MS3/MS4 = third and fourth year medical students, ABSN = Accelerated Bachelor of Science in Nursing, NP = Nurse Practitioner, PA = Physician’s Associate, DPT = Physical Therapy, MD = Doctor of Medicine

### Reasoning for preferences of fellow IPE participants

It was found that 22/44 (50.0%) MS1s selected their first-choice preference because the profession seemed most similar, compared with only 24/83 (28.9%) MS3s/MS4s (Table 1). Further, 35 (42.2%) of MS3/MS4s selected their first-choice preference because the profession seemed most different, compared with only 7 (15.9%) MS1s. Other responses were similar between MS1s and MS3s/MS4s. A significant difference in the reason for first-choice preference was found between MS1 and MS3/4 students (p = 0.006).

## Discussion

In this novel, work-in-progress study, we show that medical students participating in the Duke IPE Clinic have specific desires for who they would like to interact with during this clinical experience. Furthermore, these preferences and the underlying motivation for this choice differed by level of training.

Interestingly, when looking at how each individual profession was ranked by the MS1s compared to MS3/MS4s, we did not find any statistically significant differences. However, MS1s preferred to work with PA students the most, and MS3/MS4s preferred to work with pharmacy students the most. When grouped by similarity in profession, this resulted in a statistically significant difference. Specifically, MS1s were more likely to want to work with students from professions that are generally perceived to be more similar clinical providers (NP and PA), while MS3s/MS4s were more likely to prefer working with students from professions that are perceived as more dissimilar to the medical doctors (DPT, pharmacy, and ABSN). The likely reason for the lack of statistical significance in the initial analysis is that combining these into ‘more similar’ and ‘less similar’ categories contributed to the power to detect differences. This is further confirmed by the fact that more MS1s selected that they wanted to work with students in similar health professions as their reasoning for their first-choice preference than MS3s/MS4s, and more MS3s/MS4s answered that they wanted to work with students in different health professions.

While several studies have explored different aspects of student preference for interprofessional education, few have identified how this differs among medical students at varying levels of training. Two prior studies reported that students from various healthcare professional programs, including medical students, had perceptions of IPE that differed by year of study [15, 16]. Specifically, these differences were found in their recognition in how they thought about their roles and responsibilities, which was more solidified in later years. This was thought to likely be due to their increased clinical exposure. While differences in clinical exposure could explain the differences we found in our study, we also theorized that MS1s preferred to work with students from similar professions because they wanted to learn more about their own role and how to distinguish themselves in healthcare as future doctors. In fact, prior studies have demonstrated that implementing IPE experiences early in the curriculum is likely to have an impact on students’ ability to assume their given roles and responsibilities, and is beneficial for preventing interprofessional discrimination [17, 18]. Understanding their own professional role in the healthcare team is of great importance and lack of clarity about these roles may lead to disharmonious relationships with other health professionals [19].

In contrast, it is possible that MS3s/MS4s wanted to learn how to more expertly collaborate with different professions to which they may not have had prior exposure. As medical students advance in training, they have more insight into the importance of teamwork [17] and may have a stronger desire to understand how to work and communicate effectively with their interprofessional colleagues to succeed.

The present study has several strengths and limitations. This is the first study to our knowledge that investigates medical student preferences for interprofessional collaborators in an interprofessional education setting. Although our sample size was small, we were still able to detect a statistically significant difference in the similarity of first choice preferences. However, it only included students from one medical school, where there is a mandatory IPE clinical experience for medical students. Additionally, while participation in the IPE clinical experience was mandatory, response to our survey was voluntary. Voluntary response sampling is known to induce bias in a number of contexts [20, 21]. For example, the sample of students who responded may be those who hold strong opinions of the primary questions asked in this study, and hence, this set of volunteers is a biased sample not representing the population of medical students as a whole. Due to the de-identified nature of our data collection and the lack of comparable studies, further examination of the degree of bias in our study was not possible. The external validity and transferability of the conclusions to students in other institutions is limited, as other medical school curricula both in the United States and internationally may inherently include a different profile of health professionals and hence, have more or less exposure to the other health professions than ours and may subsequently influence who students want to work with. Further, for international curricula, the roles of different healthcare professionals may vary greatly. For example, clinically active PAs only exist in 18 countries around the world, thus they do not fit into the greater structure of the healthcare system for most of the world [22]. Finally, in this portion of the greater study, we only surveyed medical students, which only provides one perspective for how to design IPE collaborative teams. In future studies, we will conduct a more thorough investigation into the perspective of other health professions students to determine how their preferences can contribute to curricular design in the interprofessional setting. Representation of these perspectives is critical, as prior studies have shown that there have been many studies that demonstrate that other health professions view their relationships with doctors more negatively than the doctors themselves, who report a more positive perception of their relationship [23, 24].

## Conclusions

In this preliminary study, we show that medical students have varied preferences for who they work with in the IPE Clinic. Students earlier in training have a greater interest in working with professions they view as similar whereas students later in training would prefer to seek out and work with professions that they view as more different than theirs. This information is important for designing and implementing educational IPE opportunities, as it is well-established that students exhibit preferences for learning approaches and this can influence student learning [25, 26]. In 2021, the Interprofessional Education Collaborative began a formal review of the core competencies that were most recently renewed in 2016 (IPEC 2016). These core competencies frame the national dialogue on how implementing interprofessional education and collaborative practice can improve patient care and health outcomes. As we continue to develop research on student preferences in IPE, our hope is that we can contribute to the development of theories on best practice for implementing these educational opportunities for health professions students.

## Data Availability

The datasets used and analyzed during the current study are available from the corresponding author on reasonable request.

## Declarations

All methods were carried out in accordance with relevant guidelines and regulations. Informed consent was obtained from all subjects.

## Abbreviations

IPE: Interprofessional Education
DPT: Doctor of Physical Therapy
ABSN: Accelerated Bachelor of Science in Nursing
NP: Nurse Practitioner
PA: Physician’s Associate
MS1s: First–Year Medical Students
MS3s/MS4s: Third–and Fourth–Year Medical Students
